# Biology of Bone Tissue: Structure, Function, and Factors That Influence Bone Cells

**DOI:** 10.1155/2015/421746

**Published:** 2015-07-13

**Authors:** Rinaldo Florencio-Silva, Gisela Rodrigues da Silva Sasso, Estela Sasso-Cerri, Manuel Jesus Simões, Paulo Sérgio Cerri

**Affiliations:** ^1^Department of Morphology and Genetics, Laboratory of Histology and Structural Biology, Federal University of São Paulo, 04023-900 São Paulo, SP, Brazil; ^2^Department of Morphology, Laboratory of Histology and Embryology, Dental School, Universidade Estadual Paulista (UNESP), 14801-903 Araraquara, SP, Brazil

## Abstract

Bone tissue is continuously remodeled through the concerted actions of bone cells, which include bone resorption by osteoclasts and bone formation by osteoblasts, whereas osteocytes act as mechanosensors and orchestrators of the bone remodeling process. This process is under the control of local (e.g., growth factors and cytokines) and systemic (e.g., calcitonin and estrogens) factors that all together contribute for bone homeostasis. An imbalance between bone resorption and formation can result in bone diseases including osteoporosis. Recently, it has been recognized that, during bone remodeling, there are an intricate communication among bone cells. For instance, the coupling from bone resorption to bone formation is achieved by interaction between osteoclasts and osteoblasts. Moreover, osteocytes produce factors that influence osteoblast and osteoclast activities, whereas osteocyte apoptosis is followed by osteoclastic bone resorption. The increasing knowledge about the structure and functions of bone cells contributed to a better understanding of bone biology. It has been suggested that there is a complex communication between bone cells and other organs, indicating the dynamic nature of bone tissue. In this review, we discuss the current data about the structure and functions of bone cells and the factors that influence bone remodeling.

## 1. Introduction

Bone is a mineralized connective tissue that exhibits four types of cells: osteoblasts, bone lining cells, osteocytes, and osteoclasts [1, 2]. Bone exerts important functions in the body, such as locomotion, support and protection of soft tissues, calcium and phosphate storage, and harboring of bone marrow [3, 4]. Despite its inert appearance, bone is a highly dynamic organ that is continuously resorbed by osteoclasts and neoformed by osteoblasts. There is evidence that osteocytes act as mechanosensors and orchestrators of this bone remodeling process [5–8]. The function of bone lining cells is not well clear, but these cells seem to play an important role in coupling bone resorption to bone formation [9].

Bone remodeling is a highly complex process by which old bone is replaced by new bone, in a cycle comprised of three phases: (1) initiation of bone resorption by osteoclasts, (2) the transition (or reversal period) from resorption to new bone formation, and (3) the bone formation by osteoblasts [10, 11]. This process occurs due to coordinated actions of osteoclasts, osteoblasts, osteocytes, and bone lining cells which together form the temporary anatomical structure called basic multicellular unit (BMU) [12–14].

Normal bone remodeling is necessary for fracture healing and skeleton adaptation to mechanical use, as well as for calcium homeostasis [15]. On the other hand, an imbalance of bone resorption and formation results in several bone diseases. For example, excessive resorption by osteoclasts without the corresponding amount of nerformed bone by osteoblasts contributes to bone loss and osteoporosis [16], whereas the contrary may result in osteopetrosis [17]. Thus, the equilibrium between bone formation and resorption is necessary and depends on the action of several local and systemic factors including hormones, cytokines, chemokines, and biomechanical stimulation [18–20].

Recent studies have shown that bone influences the activity of other organs and the bone is also influenced by other organs and systems of the body [21], providing new insights and evidencing the complexity and dynamic nature of bone tissue.

In this review we will address the current data about bone cells biology, bone matrix, and the factors that influence the bone remodeling process. Moreover, we will briefly discuss the role of estrogen on bone tissue under physiological and pathological conditions.

## 2. Bone Cells

### 2.1. Osteoblasts

**Figure 1:**
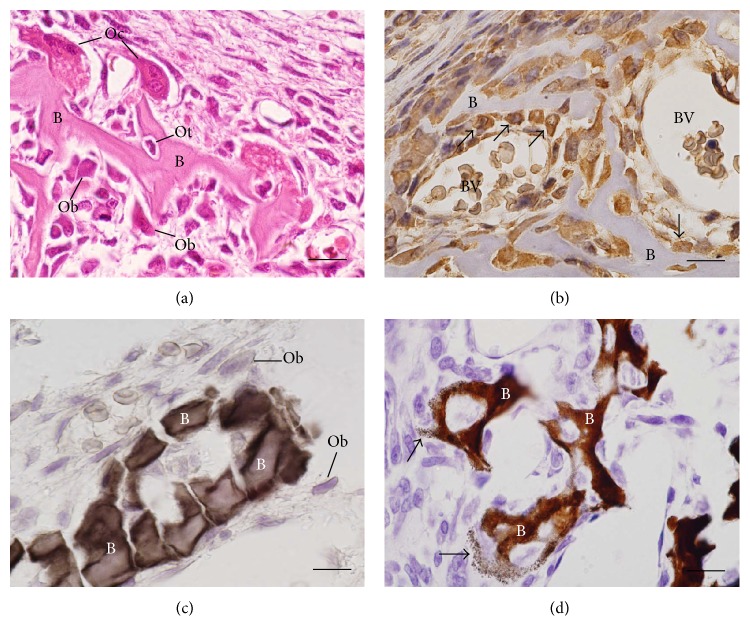
(a)–(d) Light micrographs of portions of alveolar bone of rats. (a) HE-stained section showing a portion of a bony trabecula (B). Polarized osteoblasts (Ob) and giant multinucleated osteoclasts (Oc) are observed in the bone surface; osteocyte (Ot) surrounding bone matrix is also observed. (b) Section subjected to immunohistochemistry for osteocalcin detection and counterstained with hematoxylin. Note osteocalcin-positive osteoblasts (arrows) on the surface of a bony trabecula (B). BV: blood vessel. (c) Undecalcified section subjected to the Gomori method for the detection of alkaline phosphatase, evidencing a portion of bone matrix (B) positive to the alkaline phosphatase (in brown/black). Ob: osteoblasts. (d) Undecalcified section subjected to the von Kossa method for calcium detection (brown/dark color). von Kossa-positive bone matrix (B) is observed; some positive granules (arrow) can also be observed on the surface of the bone trabeculae. Scale bar: 15 *μ*m.

**Figure 2:**
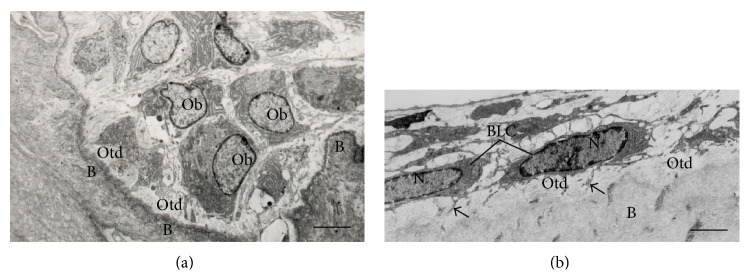
Electron micrographs of portions of alveolar bone of rats. (a) Oteoblasts exhibiting abundant rough endoplasmic reticulum are observed adjacent to the bone (B) surface. A layer of bundles of collagen fibrils situated between osteoblasts (Ob) and calcified bone surface (B) constitutes the osteoid (Otd). Scale bar: 2.7 *μ*m. (b) Bone lining cells (BLC) exhibiting scarce cytoplasm are situated on the osteoid surface (Otd). Bone lining cells (BLC) extend some thin cytoplasmic projections (arrows) towards the osteoid (Otd). Scale bar: 2 *µ*m. N: nucleus.

Osteoblasts are cuboidal cells that are located along the bone surface comprising 4–6% of the total resident bone cells and are largely known for their bone forming function [22]. These cells show morphological characteristics of protein synthesizing cells, including abundant rough endoplasmic reticulum and prominent Golgi apparatus, as well as various secretory vesicles [22, 23]. As polarized cells, the osteoblasts secrete the osteoid toward the bone matrix [24] (Figures 1(a), 1(b), and 2(a)).

Osteoblasts are derived from mesenchymal stem cells (MSC). The commitment of MSC towards the osteoprogenitor lineage requires the expression of specific genes, following timely programmed steps, including the synthesis of bone morphogenetic proteins (BMPs) and members of the Wingless (Wnt) pathways [25]. The expressions of Runt-related transcription factors 2, Distal-less homeobox 5 (Dlx5), and osterix (Osx) are crucial for osteoblast differentiation [22, 26]. Additionally,* Runx2* is a master gene of osteoblast differentiation, as demonstrated by the fact that Runx2-null mice are devoid of osteoblasts [26, 27].* Runx2* has demonstrated to upregulate osteoblast-related genes such as* ColIA1*,* ALP*,* BSP*,* BGLAP*, and* OCN* [28].

Once a pool of osteoblast progenitors expressing* Runx2* and* ColIA1* has been established during osteoblast differentiation, there is a proliferation phase. In this phase, osteoblast progenitors show alkaline phosphatase (ALP) activity, and are considered preosteoblasts [22]. The transition of preosteoblasts to mature osteoblasts is characterized by an increase in the expression of Osx and in the secretion of bone matrix proteins such as osteocalcin (OCN), bone sialoprotein (BSP) I/II, and collagen type I. Moreover, the osteoblasts undergo morphological changes, becoming large and cuboidal cells [26, 29–31].

There is evidence that other factors such as fibroblast growth factor (FGF), microRNAs, and connexin 43 play important roles in the osteoblast differentiation [32–35]. FGF-2 knockout mice showed a decreased bone mass coupled to increase of adipocytes in the bone marrow, indicating the participation of FGFs in the osteoblast differentiation [34]. It has also been demonstrated that FGF-18 upregulates osteoblast differentiation in an autocrine mechanism [36]. MicroRNAs are involved in the regulation of gene expression in many cell types, including osteoblasts, in which some microRNAs stimulate and others inhibit osteoblast differentiation [37, 38]. Connexin 43 is known to be the main connexin in bone [35]. The mutation in the gene encoding connexin 43 impairs osteoblast differentiation and causes skeletal malformation in mouse [39].

The synthesis of bone matrix by osteoblasts occurs in two main steps: deposition of organic matrix and its subsequent mineralization (Figures 1(b)–1(d)). In the first step, the osteoblasts secrete collagen proteins, mainly type I collagen, noncollagen proteins (OCN, osteonectin, BSP II, and osteopontin), and proteoglycan including decorin and biglycan, which form the organic matrix. Thereafter, mineralization of bone matrix takes place into two phases: the vesicular and the fibrillar phases [40, 41]. The vesicular phase occurs when portions with a variable diameter ranging from 30 to 200 nm, called matrix vesicles, are released from the apical membrane domain of the osteoblasts into the newly formed bone matrix in which they bind to proteoglycans and other organic components. Because of its negative charge, the sulphated proteoglycans immobilize calcium ions that are stored within the matrix vesicles [41, 42]. When osteoblasts secrete enzymes that degrade the proteoglycans, the calcium ions are released from the proteoglycans and cross the calcium channels presented in the matrix vesicles membrane. These channels are formed by proteins called annexins [40].

On the other hand, phosphate-containing compounds are degraded by the ALP secreted by osteoblasts, releasing phosphate ions inside the matrix vesicles. Then, the phosphate and calcium ions inside the vesicles nucleate, forming the hydroxyapatite crystals [43]. The fibrillar phase occurs when the supersaturation of calcium and phosphate ions inside the matrix vesicles leads to the rupture of these structures and the hydroxyapatite crystals spread to the surrounding matrix [44, 45].

**Figure 3:**
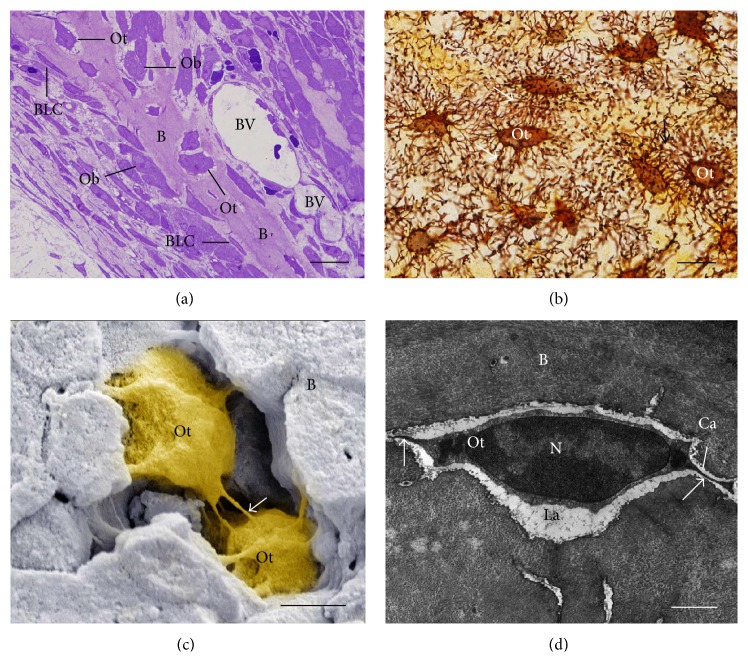
Light (a and b) and electron micrographs of portions of alveolar bone rats. (a) a semithin section stained with toluidine blue showing a portion of a bony trabecula (B). Osteoblasts (Ob) and bone lining cells (BLC) are present on bone surface while osteocytes (Ot) are observed entrapped in the bone matrix. BV: blood vessels. Scale bar: 15 *μ*m. (b) Section subjected to the silver impregnation method. Note the cytoplasmic processes (arrows) of the osteocytes (Ot) connecting them with each other. Scale bar: 15 *μ*m. (c) Scanning electron micrograph showing two osteocytes (Ot) surrounded by bone matrix (B). Note that the cytoplasmic processes (arrows) are observed between the osteocytes (Ot) forming an interconnected network. Scale bar: 2 *μ*m. (d) Transmission electron micrograph showing a typical osteocyte (Ot) inside a lacuna (La) in the bone matrix (B), with its cytoplasmic processes (arrows) inside the canaliculi (Ca). Scale bar: 2 *μ*m. N: nucleus.

Mature osteoblasts appear as a single layer of cuboidal cells containing abundant rough endoplasmic reticulum and large Golgi complex (Figures 2(a) and 3(a)). Some of these osteoblasts show cytoplasmic processes towards the bone matrix and reach the osteocyte processes [46]. At this stage, the mature osteoblasts can undergo apoptosis or become osteocytes or bone lining cells [47, 48]. Interestingly, round/ovoid structures containing dense bodies and TUNEL-positive structures have been observed inside osteoblast vacuoles. These findings suggest that besides professional phagocytes, osteoblasts are also able to engulf and degrade apoptotic bodies during alveolar bone formation [49].

### 2.2. Bone Lining Cells

Bone lining cells are quiescent flat-shaped osteoblasts that cover the bone surfaces, where neither bone resorption nor bone formation occurs [50]. These cells exhibit a thin and flat nuclear profile; its cytoplasm extends along the bone surface and displays few cytoplasmic organelles such as profiles of rough endoplasmic reticulum and Golgi apparatus [50] (Figure 2(b)). Some of these cells show processes extending into canaliculi, and gap junctions are also observed between adjacent bone lining cells and between these cells and osteocytes [50, 51].

The secretory activity of bone lining cells depends on the bone physiological status, whereby these cells can reacquire their secretory activity, enhancing their size and adopting a cuboidal appearance [52]. Bone lining cells functions are not completely understood, but it has been shown that these cells prevent the direct interaction between osteoclasts and bone matrix, when bone resorption should not occur, and also participate in osteoclast differentiation, producing osteoprotegerin (OPG) and the receptor activator of nuclear factor kappa-B ligand (RANKL) [14, 53]. Moreover, the bone lining cells, together with other bone cells, are an important component of the BMU, an anatomical structure that is present during the bone remodeling cycle [9].

### 2.3. Osteocytes

Osteocytes, which comprise 90–95% of the total bone cells, are the most abundant and long-lived cells, with a lifespan of up to 25 years [54]. Different from osteoblasts and osteoclasts, which have been defined by their respective functions during bone formation and bone resorption, osteocytes were earlier defined by their morphology and location. For decades, due to difficulties in isolating osteocytes from bone matrix led to the erroneous notion that these cells would be passive cells, and their functions were misinterpreted [55]. The development of new technologies such as the identification of osteocyte-specific markers, new animal models, development of techniques for bone cell isolation and culture, and the establishment of phenotypically stable cell lines led to the improvement of the understanding of osteocyte biology. In fact, it has been recognized that these cells play numerous important functions in bone [8].

The osteocytes are located within lacunae surrounded by mineralized bone matrix, wherein they show a dendritic morphology [15, 55, 56] (Figures 3(a)–3(d)). The morphology of embedded osteocytes differs depending on the bone type. For instance, osteocytes from trabecular bone are more rounded than osteocytes from cortical bone, which display an elongated morphology [57].

Osteocytes are derived from MSCs lineage through osteoblast differentiation. In this process, four recognizable stages have been proposed: osteoid-osteocyte, preosteocyte, young osteocyte, and mature osteocyte [54]. At the end of a bone formation cycle, a subpopulation of osteoblasts becomes osteocytes incorporated into the bone matrix. This process is accompanied by conspicuous morphological and ultrastructural changes, including the reduction of the round osteoblast size. The number of organelles such as rough endoplasmic reticulum and Golgi apparatus decreases, and the nucleus-to-cytoplasm ratio increases, which correspond to a decrease in the protein synthesis and secretion [58].

During osteoblast/osteocyte transition, cytoplasmic process starts to emerge before the osteocytes have been encased into the bone matrix [22]. The mechanisms involved in the development of osteocyte cytoplasmic processes are not well understood. However, the protein E11/gp38, also called podoplanin may have an important role. E11/gp38 is highly expressed in embedding or recently embedded osteocytes, similarly to other cell types with dendritic morphology such as podocytes, type II lung alveolar cells, and cells of the choroid plexus [59]. It has been suggested that E11/gp38 uses energy from GTPase activity to interact with cytoskeletal components and molecules involved in cell motility, whereby regulate actin cytoskeleton dynamics [60, 61]. Accordingly, inhibition of E11/gp38 expression in osteocyte-like MLO-Y4 cells has been shown to block dendrite elongation, suggesting that E11/gp38 is implicated in dendrite formation in osteocytes [59].

Once the stage of mature osteocyte totally entrapped within mineralized bone matrix is accomplished, several of the previously expressed osteoblast markers such as OCN, BSPII, collagen type I, and ALP are downregulated. On the other hand, osteocyte markers including dentine matrix protein 1 (DMP1) and sclerostin are highly expressed [8, 62–64].

Whereas the osteocyte cell body is located inside the lacuna, its cytoplasmic processes (up to 50 per each cell) cross tiny tunnels that originate from the lacuna space called canaliculi, forming the osteocyte lacunocanalicular system [65] (Figures 3(b)–3(d)). These cytoplasmic processes are connected to other neighboring osteocytes processes by gap junctions, as well as to cytoplasmic processes of osteoblasts and bone lining cells on the bone surface, facilitating the intercellular transport of small signaling molecules such as prostaglandins and nitric oxide among these cells [66]. In addition, the osteocyte lacunocanalicular system is in close proximity to the vascular supply, whereby oxygen and nutrients achieve osteocytes [15].

It has been estimated that osteocyte surface is 400-fold larger than that of the all Haversian and Volkmann systems and more than 100-fold larger than the trabecular bone surface [67, 68]. The cell-cell communication is also achieved by interstitial fluid that flows between the osteocytes processes and canaliculi [68]. By the lacunocanalicular system (Figure 3(b)), the osteocytes act as mechanosensors as their interconnected network has the capacity to detect mechanical pressures and loads, thereby helping the adaptation of bone to daily mechanical forces [55]. By this way, the osteocytes seem to act as orchestrators of bone remodeling, through regulation of osteoblast and osteoclast activities [15, 69]. Moreover, osteocyte apoptosis has been recognized as a chemotactic signal to osteoclastic bone resorption [70–73]. In agreement, it has been shown that during bone resorption, apoptotic osteocytes are engulfed by osteoclasts [74–76].

The mechanosensitive function of osteocytes is accomplished due to the strategic location of these cells within bone matrix. Thus, the shape and spatial arrangement of the osteocytes are in agreement with their sensing and signal transport functions, promoting the translation of mechanical stimuli into biochemical signals, a phenomenon that is called piezoelectric effect [77]. The mechanisms and components by which osteocytes convert mechanical stimuli to biochemical signals are not well known. However, two mechanisms have been proposed. One of them is that there is a protein complex formed by a cilium and its associated proteins PolyCystins 1 and 2, which has been suggested to be crucial for osteocyte mechanosensing and for osteoblast/osteocyte-mediated bone formation [78]. The second mechanism involves osteocyte cytoskeleton components, including focal adhesion protein complex and its multiple actin-associated proteins such as paxillin, vinculin, talin, and zyxin [79]. Upon mechanical stimulation, osteocytes produce several secondary messengers, for example, ATP, nitric oxide (NO), Ca^2+^, and prostaglandins (PGE_2_ and PGI_2_,) which influence bone physiology [8, 80]. Independently of the mechanism involved, it is important to mention that the mechanosensitive function of osteocytes is possible due to the intricate canalicular network, which allows the communication among bone cells.

### 2.4. Osteoclasts

**Figure 4:**
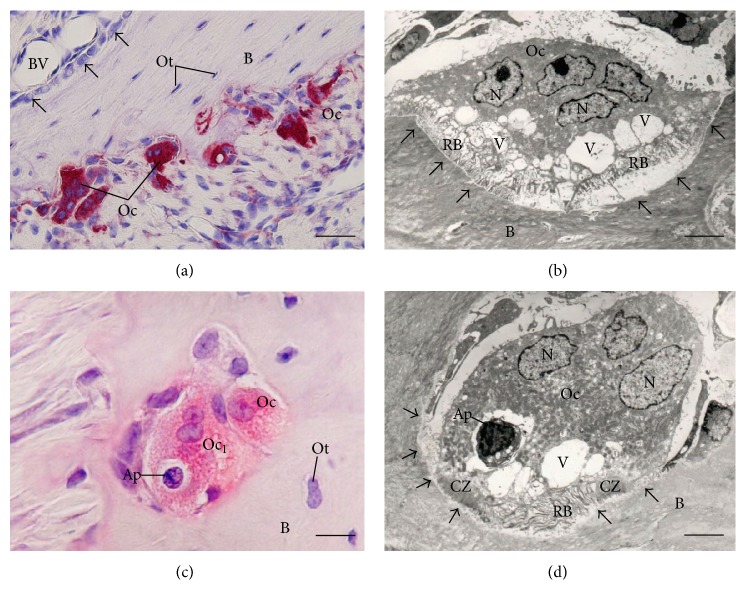
Light (a and c) and electron micrographs (b and d) of portions of alveolar bone of rats. In (a) tartrate-resistant acid phosphatase (TRAP) activity (in red color) is observed in the cytoplasm of osteoclasts (OC) adjacent to the alveolar bone (B) surface. Note that in the opposite side of the bony trabecula B is covered by large and polarized osteoblasts (Ob). Ot, osteocytes (Ot); BV: blood vessel. Bar: 40 *μ*m. (b) Multinucleated osteoclast (OC) shows evident ruffled border (RB) adjacent to the excavated bone surface (arrows). Several vacuoles (V) are observed in the cytoplasm adjacent to ruffled border (RB). N: nucleus. Bar: 4 *μ*m. (c) Portions of TRAP-positive osteoclasts (Oc and Oc_1_) are observed in a resorbing bone lacuna. A round cell (Ap) with condensed irregular blocks of chromatin, typical apoptotic cell, is observed inside a large vacuole of the Oc_1_. B: bone matrix; Ot: osteocyte. Bar: 15 *μ*m. (d) An osteoclast (Oc) showing ruffled border (RB) and clear zone (CZ) is in close juxtaposition to the excavation of the bone surface (arrows), that is, Howship lacuna. Vacuoles (V) with varied size are present next to the ruffled border (RB); one of them contains a round cell with masses of condensed chromatin (Ap), typical of cell undergoing apoptosis. B: bone matrix; N: nucleus. Bar: 3 *μ*m.

Osteoclasts are terminally differentiated multinucleated cells (Figures 4(a)–4(d)), which originate from mononuclear cells of the hematopoietic stem cell lineage, under the influence of several factors. Among these factors the macrophage colony-stimulating factor (M-CSF), secreted by osteoprogenitor mesenchymal cells and osteoblasts [81], and RANK ligand, secreted by osteoblasts, osteocytes, and stromal cells, are included [20]. Together, these factors promote the activation of transcription factors [81, 82] and gene expression in osteoclasts [83, 84].

**Figure 5:**
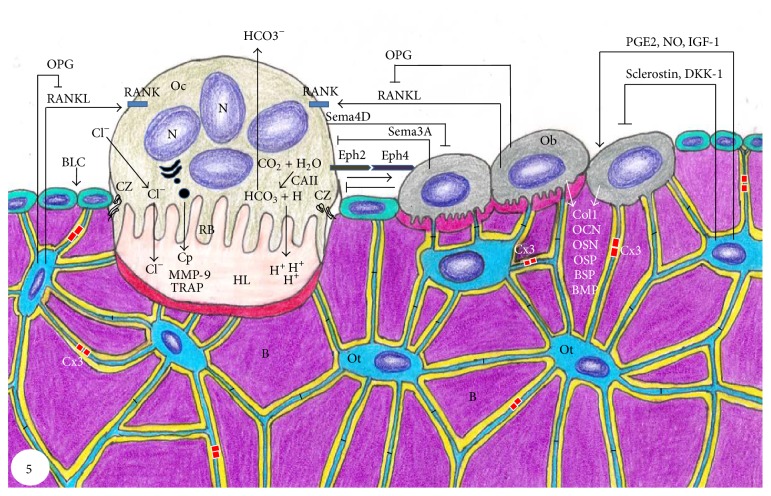
Schematic summary of bone tissue showing bone cells and the relationships among them and with bone matrix (B). Osteoclast (Oc) activation occurs after binding of RANKL to its receptor RANK, present in the membrane of osteoclast precursors. Then, osteoclast becomes polarized through its cytoskeleton reorganization; the ruffled border (RB) and clear zone (CZ) are membrane specializations observed in the portion of the osteoclast juxtaposed to the bone resorption surface, Howship lacuna (HL). Dissolution of hydroxyapatite occurs in the bone surface adjacent to the ruffled border (RF) upon its acidification due to pumping of hydrogen ions (H^+^) to the HL. H^+^ and ions bicarbonate (HCO_3_^−^) originate from the cleavage of carbonic acid (H_2_CO_3_) under the action of carbonic anhydrase II (CAII). After dissolution of mineral phase, osteoclast (Oc) releases cathepsin (Cp), matrix metalloproteinase-9 (MMP-9), and tartrate-resistant acid phosphatase (TRAP) that degrade the organic matrix. EphrinB2 (Eph2) present in osteoclast membrane binds to ephrinB4 (Eph4) in osteoblast (Ob) membrane, promoting its differentiation, whereas the reverse signaling (ephrinB4/ephrinB2) inhibits osteoclastogenesis. Sema4D produced by osteoclasts inhibits osteoblasts, while Sema3A secreted by osteoblasts inhibits osteoclasts. Osteoblasts (Ob) also produce receptor activator of nuclear factor KB (RANKL) and osteoprotegerin (OPG), which increase and decrease osteoclastogenesis, respectively. Osteoblasts (Ob) secrete collagenous (Col1) and noncollagenous proteins such as osteocalcin (OCN), osteopontin (OSP), osteonectin (OSN), bone sialoprotein (BSP), and bone morphogenetic proteins (BMP). Osteocytes (Ot) are located within lacunae surrounded by mineralized bone matrix (B). Its cytoplasmic processes cross canaliculi to make connection with other neighboring osteocytes processes by gap junctions, mainly composed by connexin 43 (Cx3), as well as to cytoplasmic processes of osteoblasts (Ob) and bone lining cells (BLC) on bone surface. RANKL secreted by osteocytes stimulates osteoclastogenesis, while prostaglandin E_2_ (PGE2), nitric oxide (NO), and insulin-like growth factor (IGF) stimulate osteoblast activity. Conversely, osteocytes produce OPG that inhibits osteoclastogenesis; moreover, osteocytes produce sclerostin and dickkopf WNT signaling pathway inhibitor (DKK-1) that decrease osteoblast activity.

M-CSF binds to its receptor (cFMS) present in osteoclast precursors, which stimulates their proliferation and inhibits their apoptosis [82, 85]. RANKL is a crucial factor for osteoclastogenesis and is expressed by osteoblasts, osteocytes, and stromal cells. When it binds to its receptor RANK in osteoclast precursors, osteoclast formation is induced [86]. On the other hand, another factor called osteoprotegerin (OPG), which is produced by a wide range of cells including osteoblasts, stromal cells, and gingival and periodontal fibroblasts [87–89], binds to RANKL, preventing the RANK/RANKL interaction and, consequently, inhibiting the osteoclastogenesis [87] (Figure 5). Thus, the RANKL/RANK/OPG system is a key mediator of osteoclastogenesis [19, 86, 89].

The RANKL/RANK interaction also promotes the expression of other osteoclastogenic factors such as NFATc1 and DC-STAMP. By interacting with the transcription factors PU.1, cFos, and MITF, NFATc1 regulates osteoclast-specific genes including* TRAP* and* cathepsin K*, which are crucial for osteoclast activity [90]. Under the influence of the RANKL/RANK interaction, NFATc1 also induces the expression of DC-STAMP, which is crucial for the fusion of osteoclast precursors [91, 92].

Despite these osteoclastogenic factors having been well defined, it has recently been demonstrated that the osteoclastogenic potential may differ depending on the bone site considered. It has been reported that osteoclasts from long bone marrow are formed faster than in the jaw. This different dynamic of osteoclastogenesis possibly could be, due to the cellular composition of the bone-site specific marrow [93].

During bone remodeling osteoclasts polarize; then, four types of osteoclast membrane domains can be observed: the sealing zone and ruffled border that are in contact with the bone matrix (Figures 4(b) and 4(d)), as well as the basolateral and functional secretory domains, which are not in contact with the bone matrix [94, 95]. Polarization of osteoclasts during bone resorption involves rearrangement of the actin cytoskeleton, in which an F-actin ring that comprises a dense continuous zone of highly dynamic podosome is formed and consequently an area of membrane that develop into the ruffled border is isolated. It is important to mention that these domains are only formed when osteoclasts are in contact with extracellular mineralized matrix, in a process which *α*_v_ *β*_3_-integrin, as well as the CD44, mediates the attachment of the osteoclast podosomes to the bone surface [96–99]. Ultrastructurally, the ruffled border is a membrane domain formed by microvilli, which is isolated from the surrounded tissue by the clear zone, also known as sealing zone. The clear zone is an area devoid of organelles located in the periphery of the osteoclast adjacent to the bone matrix [98]. This sealing zone is formed by an actin ring and several other proteins, including actin, talin, vinculin, paxillin, tensin, and actin-associated proteins such as *α*-actinin, fimbrin, gelsolin, and dynamin [95]. The *α*_v_ *β*_3_-integrin binds to noncollagenous bone matrix containing-RGD sequence such as bone sialoprotein, osteopontin, and vitronectin, establishing a peripheric sealing that delimits the central region, where the ruffled border is located [98] (Figures 4(b)–4(d)).

The maintenance of the ruffled border is also essential for osteoclast activity; this structure is formed due to intense trafficking of lysosomal and endosomal components. In the ruffled border, there is a vacuolar-type H^+^-ATPase (V-ATPase), which helps to acidify the resorption lacuna and hence to enable dissolution of hydroxyapatite crystals [20, 100, 101]. In this region, protons and enzymes, such as tartrate-resistant acid phosphatase (TRAP), cathepsin K, and matrix metalloproteinase-9 (MMP-9) are transported into a compartment called Howship lacuna leading to bone degradation [94, 101–104] (Figure 5). The products of this degradation are then endocytosed across the ruffled border and transcytosed to the functional secretory domain at the plasma membrane [7, 95].

Abnormal increase in osteoclast formation and activity leads to some bone diseases such as osteoporosis, where resorption exceeds formation causing decreased bone density and increased bone fractures [105]. In some pathologic conditions including bone metastases and inflammatory arthritis, abnormal osteoclast activation results in periarticular erosions and painful osteolytic lesions, respectively [83, 105, 106]. In periodontitis, a disease of the periodontium caused by bacterial proliferation [107, 108] induces the migration of inflammatory cells. These cells produce chemical mediators such as IL-6 and RANKL that stimulate the migration of osteoclasts [89, 109, 110]. As a result, an abnormal increased bone resorption occurs in the alveolar bone, contributing to the loss of the insertions of the teeth and to the progression of periodontitis [89, 111].

On the other hand, in osteopetrosis, which is a rare bone disease, genetic mutations that affect formation and resorption functions in osteoclasts lead to decreased bone resorption, resulting in a disproportionate accumulation of bone mass [17]. These diseases demonstrate the importance of the normal bone remodeling process for the maintenance of bone homeostasis.

Furthermore, there is evidence that osteoclasts display several other functions. For example, it has been shown that osteoclasts produce factors called clastokines that control osteoblast during the bone remodeling cycle, which will be discussed below. Other recent evidence is that osteoclasts may also directly regulate the hematopoietic stem cell niche [112]. These findings indicate that osteoclasts are not only bone resorbing cells, but also a source of cytokines that influence the activity of other cells.

### 2.5. Extracellular Bone Matrix

Bone is composed by inorganic salts and organic matrix [113]. The organic matrix contains collagenous proteins (90%), predominantly type I collagen, and noncollagenous proteins including osteocalcin, osteonectin, osteopontin, fibronectin and bone sialoprotein II, bone morphogenetic proteins (BMPs), and growth factors [114]. There are also small leucine-rich proteoglycans including decorin, biglycan, lumican, osteoaderin, and seric proteins [114–116].

The inorganic material of bone consists predominantly of phosphate and calcium ions; however, significant amounts of bicarbonate, sodium, potassium, citrate, magnesium, carbonate, fluorite, zinc, barium, and strontium are also present [1, 2]. Calcium and phosphate ions nucleate to form the hydroxyapatite crystals, which are represented by the chemical formula Ca_10_(PO_4_)_6_(OH)_2_. Together with collagen, the noncollagenous matrix proteins form a scaffold for hydroxyapatite deposition and such association is responsible for the typical stiffness and resistance of bone tissue [4].

Bone matrix constitutes a complex and organized framework that provides mechanical support and exerts essential role in the bone homeostasis. The bone matrix can release several molecules that interfere in the bone cells activity and, consequently, has a participation in the bone remodeling [117]. Once loss of bone mass alone is insufficient to cause bone fractures [118], it is suggested that other factors, including changes in the bone matrix proteins and their modifications, are of crucial importance to the understanding and prediction of bone fractures [119]. In fact, it is known that collagen plays a critical role in the structure and function of bone tissue [120].

Accordingly, it has been demonstrated that there is a variation in the concentration of bone matrix proteins with age, nutrition, disease, and antiosteoporotic treatments [119, 121, 122] which may contribute to postyield deformation and fracture of bone [119]. For instance,* in vivo* and* in vitro* studies have reported that the increase in hyaluronic acid synthesis after parathyroid hormone (PTH) treatment was related to a subsequent bone resorption [123–127] suggesting a possible relationship between hyaluronic acid synthesis and the increase in osteoclast activity.

### 2.6. Interactions between Bone Cells and Bone Matrix

As previously discussed, bone matrix does not only provides support for bone cells, but also has a key role in regulating the activity of bone cells through several adhesion molecules [117, 128]. Integrins are the most common adhesion molecules involved in the interaction between bone cells and bone matrix [129]. Osteoblasts make interactions with bone matrix by integrins, which recognize and bind to RGD and other sequences present in bone matrix proteins including osteopontin, fibronectin, collagen, osteopontin, and bone sialoprotein [130, 131]. The most common integrins present in osteoblasts are *α*_1_ *β*_1_, *α*_2_ *β*_1_, and *α*_5_ *β*_1_ [132]. These proteins also play an important role in osteoblast organization on the bone surface during osteoid synthesis [129].

On the other hand, the interaction between osteoclasts and bone matrix is essential for osteoclast function, since as previously mentioned, bone resorption occurs only when osteoclasts bind to mineralized bone surface [97]. Thus, during bone resorption osteoclasts express *α*_v_ *β*_3_ and *α*_2_ *β*_1_ integrins to interact with the extracellular matrix, in which the former bind to bone-enriched RGD-containing proteins, such as bone sialoprotein and osteopontin, whereas *β*_1_ integrins bind to collagen fibrils [133, 134]. Despite these bindings, osteoclasts are highly motile even active resorption and, as migrating cells, osteoclasts do not express cadherins. However, it has been demonstrated that cadherins provide intimate contact between osteoclast precursors and stromal cells, which express crucial growth factors for osteoclast differentiation [135].

Integrins play a mediating role in osteocyte-bone matrix interactions. These interactions are essential for the mechanosensitive function of these cells, whereby signals induced by tissue deformation are generated and amplified [136]. It is still not clear which integrins are involved, but it has been suggested that *β*_3_ and *β*_1_ integrins are involved in osteocyte-bone matrix interaction [137, 138]. These interactions occur between osteocyte body and the bone matrix of the lacuna wall as well as between canalicular wall with the osteocyte processes [137].

Only a narrow pericellular space filled by a fluid separates the osteocyte cell body and processes from a mineralized bone matrix [58]. The space between osteocyte cell body and the lacunar wall is approximately 0.5–1.0 *μ*m wide, whereas the distance between the membranes of osteocyte processes and the canalicular wall varies from 50 to 100 nm [139]. The chemical composition of the pericellular fluid has not been precisely defined. However, a diverse array of macromolecules produced by osteocytes such as osteopontin, osteocalcin, dentin matrix protein, proteoglycans, and hyaluronic acid is present [136, 140, 141].

The osteocyte and their processes are surrounded by a nonorganized pericellular matrix; delicate fibrous connections were observed within the canalicular network, termed “tethers” [139]. It has been suggested that perlecan is a possible compound of these tethers [141]. Osteocyte processes can also attach directly by the “hillocks,” which are protruding structures originating from the canalicular walls. These structures form close contacts, possibly by means of *β*_3_-integrins, with the membrane of osteocyte processes [137, 142]. Thus, these structures seem to play a key role in the mechanosensitive function of osteocytes, by sensing the fluid flux movements along with the pericellular space, provoked by mechanical load forces [143]. In addition, the fluid flux movement is also essential for the bidirectional solute transport in the pericellular space, which influences osteocyte signaling pathways and communication among bone cells [144, 145].

### 2.7. Local and Systemic Factor That Regulate Bone Homeostasis

Bone remodeling is a highly complex cycle that is achieved by the concerted actions of osteoblasts, osteocytes, osteoclasts, and bone lining cells [3]. The formation, proliferation, differentiation, and activity of these cells are controlled by local and systemic factors [18, 19]. The local factors include autocrine and paracrine molecules such as growth factors, cytokines, and prostaglandins produced by the bone cells besides factors of the bone matrix that are released during bone resorption [46, 146]. The systemic factors which are important to the maintenance of bone homeostasis include parathyroid hormone (PTH), calcitonin, 1,25-dihydroxyvitamin D_3_ (calcitriol), glucocorticoids, androgens, and estrogens [16, 147–150]. Similar to PTH, PTH related protein (PTHrP), which also binds to PTH receptor, has also been reported to influence bone remodeling [147].

Estrogen plays crucial roles for bone tissue homeostasis; the decrease in estrogen level at menopause is the main cause of bone loss and osteoporosis [16]. The mechanisms by which estrogen act on bone tissue are not completely understood. Nevertheless, several studies have shown that estrogen maintains bone homeostasis by inhibiting osteoblast and osteocyte apoptosis [151–153] and preventing excessive bone resorption. The estrogen suppresses the osteoclast formation and activity as well as induces osteoclast apoptosis [16, 76, 104, 154]. It has been suggested that estrogen decreases osteoclast formation by inhibiting the synthesis of the osteoclastogenic cytokine RANKL by osteoblasts and osteocytes. Moreover, estrogen stimulates these bone cells to produce osteoprotegerin (OPG), a decoy receptor of RANK in osteoclast, thus inhibiting osteoclastogenesis [19, 155–159]. In addition, estrogen inhibits osteoclast formation by reducing the levels of other osteoclastogenic cytokines such as IL-1, IL-6, IL-11, TNF-*α*, TNF-*β*, and M-CSF [160, 161].

Estrogen acts directly on bone cells by its estrogen receptors *α* and *β* present on these cells [162]. Moreover, it has been shown that osteoclast is a direct target for estrogen [163, 164]. Accordingly, immunoexpression of estrogen receptor *β* has been demonstrated in alveolar bone cells of estradiol-treated female rats. Moreover, the enhanced immunoexpression observed in TUNEL-positive osteoclasts indicates that estrogen participates in the control of osteoclast life span directly by estrogen receptors [163]. These findings demonstrate the importance of estrogen for the maintenance of bone homeostasis.

### 2.8. Bone Remodeling Process

The bone remodeling cycle takes place within bone cavities that need to be remodeled [165]. In these cavities, there is the formation of temporary anatomical structures called basic multicellular units (BMUs), which are comprised of a group of osteoclasts ahead forming the cutting cone and a group of osteoblasts behind forming the closing cone, associated with blood vessels and the peripheral innervation [11, 166]. It has been suggested that BMU is covered by a canopy of cells (possibly bone lining cells) that form the bone remodeling compartment (BRC) [13]. The BRC seems to be connected to bone lining cells on bone surface, which in turn are in communication with osteocytes enclosed within the bone matrix [13, 14].

The bone remodeling cycle begins with an initiation phase, which consists of bone resorption by osteoclasts, followed by a phase of bone formation by osteoblasts but between these two phases, there is a transition (or reversal) phase. The cycle is completed by coordinated actions of osteocytes and bone lining cells [10, 11]. In the initiation phase, under the action of osteoclastogenic factors including RANKL and M-CSF, hematopoietic stem cells are recruited to specific bone surface areas and differentiate into mature osteoclasts that initiate bone resorption [167, 168].

It is known that during bone remodeling cycle, there are direct and indirect communications among bone cells in a process called coupling mechanism, which include soluble coupling factors stored in bone matrix that would be released after osteoclast bone resorption [169]. For instance, factors such as insulin-like growth factors (IGFs), transforming growth factor *β* (TGF-*β*), BMPs, FGF, and platelet-derived growth factor (PDGF) seem to act as coupling factors, since they are stored in bone matrix and released during bone resorption [170]. This idea is supported by genetic studies in humans and mice as well as by pharmacological studies [105, 171].

Recently, it has been suggested that another category of molecules called semaphorins is involved in the bone cell communication during bone remodeling [146]. During the initial phase, osteoblast differentiation and activity must be inhibited, in order to completely remove the damaged or aged bone. The osteoclasts express a factor called semaphorin4D (Sema4D) that inhibits bone formation during bone resorption [172]. Semaphorins comprise a large family of glycoproteins which are not only membrane-bound but also exist as soluble forms that are found in a wide range of tissues and shown to be involved in diverse biological processes such as immune response, organogenesis, cardiovascular development, and tumor progression [172, 173]. In bone, it has been suggested that semaphorins are also involved in cell-cell communication between osteoclasts and osteoblasts during the bone remodeling cycle [174–176].

Sema4D expressed in osteoclasts binds to its receptor (Plexin-B1) present in osteoblasts and inhibits IGF-1 pathway, essential for osteoblast differentiation [172], suggesting that osteoclasts suppress bone formation by expressing Sema4D. Conversely, another member of semaphorin family (Sema3A) has been found in osteoblasts and is considered an inhibitor of osteoclastogenesis [177]. Thus, during the bone remodeling cycle, osteoclasts inhibit bone formation by expressing Sema4D, in order to initiate bone resorption, whereas osteoblasts express Sema3A that suppresses bone resorption, prior to bone formation [146] (Figure 5).

Recent studies also suggest the existence of other factors involved in the coupling mechanism during the bone remodeling cycle. One of these factors is ephrinB2, a membrane-bound molecule expressed in mature osteoclasts, which bind to ephrinB4, found in the plasma membrane of osteoblasts. The ephrinB2/ephrinB4 binding transduces bidirectional signals, which promote osteoblast differentiation, whereas the reverse signaling (ephrinB4/ephrinB2) inhibits osteoclastogenesis [178] (Figure 5). These findings suggest that ephrinB2/ephrinB4 pathway may be involved in the ending of bone resorption and induces osteoblast differentiation in the transition phase [178].

In addition, it has been shown that ephrinB2 is also expressed in osteoblasts [179]. Furthermore, mature osteoclasts secrete a number of factors that stimulate osteoblast differentiation such as the secreted signaling molecules Wnt10b, BMP6, and the signaling sphingolipid, sphingosine-1-phosphate [180]. These findings suggest a highly complex mechanism of ephrins and the involvement of other factors in osteoclast/osteoblast communication during the bone remodeling cycle. On the other hand, despite the studies reporting the involvement of semaphorins and ephrins on osteoclast/osteoblast communication, the direct contact between mature osteoblasts and osteoclasts has not been demonstrated* in vivo* and it is still controversial.

Besides osteoclasts and osteoblasts, it has been demonstrated that osteocytes play key roles during the bone remodeling cycle [8]. In fact, under the influence of several factors, the osteocytes act as orchestrators of the bone remodeling process, producing factors that influence osteoblast and osteoclast activities [55] (Figure 5). For example, mechanical loading stimulates osteocyte to produce factors that exert anabolic action on bone such as PGE_2_, prostacyclin (PGI_2_), NO, and IGF-1 [181–184]. On the other hand, mechanical unloading downregulates anabolic factors and stimulates osteocytes to produce sclerostin and DKK-1, which are inhibitors of osteoblast activity [185–188], as well as specific factors that stimulate local osteoclastogenesis [189]. Sclerostin is a product of the* SOST* gene and is known to be a negative regulator of bone formation, by antagonizing in osteoblasts the actions of Lrp5, a key receptor of the Wnt/*β*-catenin signaling pathway [63].

Osteocyte apoptosis has been shown to act as a chemotactic signal for local osteoclast recruitment [70, 150, 152, 190, 191]. Accordingly, it has been reported that osteoclasts engulf apoptotic osteocytes [74, 75, 192], suggesting that osteoclasts are able to remove dying osteocytes and/or osteoblasts from a remodeling site (Figures 4(c) and 4(d)). Moreover, it is reported that the osteoclastogenic factors is also produced by viable osteocytes nearby the dying osteocytes [193]. There is evidence that osteocytes act as the main source of RANKL to promote osteoclastogenesis [167, 168], although this factor has also been demonstrated to be produced by other cell types such as stromal cells [194], osteoblasts, and fibroblasts [88, 89].

Thus, there are still uncertainties about the precise osteoclastogenesis-stimulating factors produced by osteocytes. Recent reviews have focused on some molecules that may be candidates for signaling between osteocyte apoptosis and osteoclastogenesis [72, 73]. For instance, in bones subjected to fatigue loading, viable osteocytes near the apoptotic ones express, besides high RANKL/OPG ratio, increased levels of vascular endothelial growth factor (VEGF) and monocyte chemoattractant protein-1 (CCL2) promoting an increase in local osteoclastogenesis [194, 195]. It has been suggested that osteocytes act as the main source of RANKL to promote osteoclastogenesis [166, 167]. In addition, an increase in RANKL/OPG ratio expressed by osteocytes was also observed in connexin43-deficient rats, suggesting that a disruption in cell-to-cell communication between osteocytes may induce the release of local proosteoclastogenic cytokines [33, 196, 197]. High mobility group box protein 1 (HMGB1) [198–200] and M-CSF [201] have also been suggested to be produced by osteocytes that stimulate osteoclast recruitment during bone remodeling [72, 73]. Thus, future studies are required to address this issue.

### 2.9. Endocrine Functions of Bone Tissue

The classical functions of bone tissue, besides locomotion, include support and protection of soft tissues, calcium, and phosphate storage and harboring of bone marrow. Additionally, recent studies have focused on the bone endocrine functions which are able to affect other organs [202]. For instance, osteocalcin produced by osteoblasts has been shown to act in other organs [203]. Osteocalcin can be found in two different forms: carboxylated and undercarboxylated. The carboxylated form has high affinity to the hydroxyapatite crystals, remaining into bone matrix during its mineralization. The undercarboxylated form shows lower affinity to minerals, due to acidification of bone matrix during osteoclast bone resorption, and then it is ferried by the bloodstream, reaching other organs [204, 205]. It has been shown that the undercarboxylated osteocalcin has some effects in pancreas, adipose tissue, testis, and the nervous system. In the pancreas, osteocalcin acts as a positive regulator of pancreatic insulin secretion and sensitivity as well as for the proliferation of pancreatic *β*-cells [110]. In the adipose tissue, osteocalcin stimulates adiponectin gene expression that in turn enhances insulin sensitivity [204]. In the testis, osteocalcin can bind to a specific receptor in Leydig cells and enhances testosterone synthesis and, consequently, increases fertility [206]. Osteocalcin also stimulates the synthesis of monoamine neurotransmitters in the hippocampus and inhibits gamma-aminobutyric acid (GABA) synthesis, improving learning and memory skills [207].

Another endocrine function of bone tissue is promoted by osteocytes. These cells are able to regulate phosphate metabolism by the production of FGF23, which acts on other organs including parathyroid gland and kidneys to reduce the circulating levels of phosphates [208, 209]. Osteocytes also act on the immune system by modifying the microenvironment in primary lymphoid organs and thereby influencing lymphopoiesis [210]. Not only osteocyte but also osteoblast and osteoclast activities are known to influence the immune system, mainly upon bone inflammatory destruction. Indeed, the discovery of communication interplay between skeletal and immune systems led to a new field of study called osteoimmunology [211].

## 3. Conclusions

The knowledge of the structural, molecular, and functional biology of bone is essential for the better comprehension of this tissue as a multicellular unit and a dynamic structure that can also act as an endocrine tissue, a function still poorly understood.* In vitro* and* in vivo* studies have demonstrated that bone cells respond to different factors and molecules, contributing to the better understanding of bone cells plasticity. Additionally, bone matrix integrins-dependent bone cells interactions are essential for bone formation and resorption. Studies have addressed the importance of the lacunocanalicular system and the pericellular fluid, by which osteocytes act as mechanosensors, for the adaptation of bone to mechanical forces. Hormones, cytokines, and factors that regulate bone cells activity, such as sclerostin, ephrinB2, and semaphoring, have played a significant role in the bone histophysiology under normal and pathological conditions. Thus, such deeper understanding of the dynamic nature of bone tissue will certainly help to manage new therapeutic approaches to bone diseases.
